# Preliminary Analytical Evaluation of the Automated Cobas i601 LC-MS/MS System for Immunosuppressant Quantification in a Clinical Laboratory

**DOI:** 10.21627/rtbk5y18

**Published:** 2026-05-19

**Authors:** Mariana Serres-Gómez, María Gemma Serrano Olmedo, María Luisa González-Casaus, Belén Fernández-Puntero, Pilar Fernández-Calle, Lucía Díaz García, Ana Rejas Muslera, Jon Sánchez-Munárriz, Antonio Buño Soto

**Affiliations:** Department of Laboratory Medicine, La Paz University Hospital, Madrid, 28046, Spain; IdiPaz-Hospital La Paz Institute for Health Research, Madrid, 28046, Spain; Department of Laboratory Medicine, La Paz University Hospital, Paseo de la Castellana 261, Madrid, 28046, Spain; Department of Laboratory Medicine, La Paz University Hospital, Madrid, 28046, Spain; IdiPaz-Hospital La Paz Institute for Health Research, Madrid, 28046, Spain; Department of Laboratory Medicine, La Paz University Hospital, Madrid, 28046, Spain; IdiPaz-Hospital La Paz Institute for Health Research, Madrid, 28046, Spain; Department of Laboratory Medicine, La Paz University Hospital, Madrid, 28046, Spain; Department of Laboratory Medicine, La Paz University Hospital, Madrid, 28046, Spain; IdiPaz-Hospital La Paz Institute for Health Research, Madrid, 28046, Spain; IdiPaz-Hospital La Paz Institute for Health Research, Madrid, 28046, Spain; Department of Clinical Pharmacology, La Paz University Hospital, Madrid, 28046, Spain; Department of Laboratory Medicine, La Paz University Hospital, Madrid, 28046, Spain; Department of Laboratory Medicine, 12 de Octubre University Hospital, Madrid, 28041, Spain; Department of Laboratory Medicine, La Paz University Hospital, Madrid, 28046, Spain; IdiPaz-Hospital La Paz Institute for Health Research, Madrid, 28046, Spain

**Keywords:** Therapeutic drug monitoring, immunosuppressants, liquid chromatography–tandem mass spectrometry, automation, immunoassay

## Abstract

**INTRODUCTION:**

Therapeutic drug monitoring (TDM) of immunosuppressive drugs is essential to maintain concentrations within narrow therapeutic windows and minimize toxicity. Automated liquid chromatography–tandem mass spectrometry (LC–MS/MS) platforms can combine the analytical specificity of mass spectrometry with workflow efficiency suitable for routine laboratories.

**OBJECTIVES:**

To preliminarily evaluate the analytical performance of the Cobas i601 automated LC–MS/MS system for quantifying tacrolimus, cyclosporine, everolimus, and sirolimus and to compare results with routine immunoassays and an LC–MS/MS method for sirolimus.

**METHODS:**

Imprecision, bias, and linearity were assessed according to the Clinical Laboratory Standards Institute (CLSI) EP10-A3 standard using quality-control materials. Method comparison and bias estimation versus the Dimension EXL 200 immunoassays and an LC–MS/MS technique for sirolimus followed the CLSI EP09-A3 standard. Regression analyses and predicted biases at therapeutic range concentrations were compared with allowable limits.

**RESULTS:**

The Cobas i601 showed good precision and linearity, meeting manufacturer specifications. The highest coefficients of variations for tacrolimus, everolimus, cyclosporine, and sirolimus were 4.0%, 4.7%, 3.6%, and 4.8%, and the maximum EP10-A3 biases were −7.4%, −3.9%, 4.7%, and 5.4%, respectively. In the method comparison, tacrolimus, everolimus, and cyclosporine showed substantial negative biases versus immunoassays (−22.7%, −38.9%, and −37.5%), exceeding allowable limits. Sirolimus exhibited smaller positive bias relative to the LC–MS/MS method (12.5%). Interchangeability between the methodologies cannot be assumed.

**CONCLUSION:**

The Cobas i601 provides reliable automated LC–MS/MS quantification of immunosuppressants, with good precision and linearity. Method-dependent biases highlight the need for careful interpretation during technology transitions. The Cobas i601 shows strong potential for routine LC–MS/MS implementation in TDM in clinical laboratories.

## Non-Specialist Summary

This study evaluated the Cobas i601, an automated system for measuring immunosuppressive drugs in blood. The system showed high precision and reliability. Some differences were observed compared with the tests currently used, highlighting the need for careful interpretation when changing methods. Overall, the Cobas i601 could improve patient care by providing more accurate drug measurements while simplifying laboratory workflow.

## 1. Introduction

Therapeutic drug monitoring (TDM) of immunosuppressive drugs plays a crucial role in solid organ transplantation and autoimmune diseases, where maintaining drug concentrations within narrow therapeutic windows is essential to prevent graft rejection and minimize toxicity [1]. Drugs such as tacrolimus, cyclosporine, sirolimus, and everolimus exhibit high pharmacokinetic variability due to factors including metabolism via CYP3A isoenzymes, drug–drug interactions, and genetic polymorphisms [2]. Consequently, precise and reliable quantification of these compounds in whole blood is a key requirement for effective patient management. Over the past decades, immunoassays have been the predominant analytical approach for TDM in routine laboratories, offering simplicity and high throughput. However, they are susceptible to cross-reactivity with metabolites or structurally related compounds, which can result in significant positive bias and misclassification of patient results [3,4]. By contrast, liquid chromatography–tandem mass spectrometry (LC–MS/MS) offers superior analytical specificity and sensitivity by directly quantifying the parent compound and is considered the reference method for immunosuppressant TDM [5]. Nonetheless, its widespread use in routine settings has been constrained by time-consuming sample preparation, longer turnaround times, the need for skilled personnel, and high operating costs.

The introduction of fully automated LC–MS/MS systems has the potential to transform clinical diagnostics by merging the superior analytical performance of mass spectrometry with the operational efficiency, reproducibility, and standardization demanded in high-throughput clinical laboratories. Previous efforts to bring automated LC–MS/MS into routine clinical laboratories have included the introduction of the Thermo Scientific Cascadion™ SM Clinical Analyzer. Several studies reported its analytical performance and usability for applications such as vitamin D and immunosuppressant drug monitoring, demonstrating the feasibility of automated LC–MS/MS in routine settings [6–8]. However, this analyzer was discontinued in 2023. In this framework, Roche introduced in early 2025 the Cobas® Mass Spec solution, comprising the Cobas® i601 analyzer, a fully automated and integrated LC–MS/MS platform. This system is designed to operate with standardized Ionify reagent kits, covering a wide spectrum of clinical applications such as immunosuppressant monitoring, antibiotics, antiepileptics, steroid hormones, and vitamin D. Among these, immunosuppressive drug quantification represents a particularly relevant field given the narrow therapeutic windows of these compounds and the clinical consequences of inaccurate measurement. Preliminary evaluation of the analyzer prototype performed in Germany demonstrated good system functionality and practicability, operating under random access conditions [9]. The authors estimated that traditional LC–MS/MS workflows involving manual sample preparation and batch analysis would have required approximately ten times longer processing and turnaround times, supporting the applicability of this system for incorporation into routine clinical laboratories with high sample volumes [9].

Following installation of a Cobas i601 automated LC–MS/MS system in our tertiary hospital, the first center in Spain to incorporate this platform, a pilot project was carried out using an immunosuppressant reagent kit that includes tacrolimus, cyclosporine, everolimus, and sirolimus. The aims of the present study were: 1) to perform a preliminary evaluation of imprecision, bias, and linearity according to the CLSI EP10-A3 protocol; and 2) to assess method comparison and bias versus our routine immunoassay analyzer and a manual LC–MS/MS technique for sirolimus, following the EP09-A3 protocol.

## 2. Materials and Methods

### 2.1. Analyzers and Reagents

The automated LC-MS/MS system Cobas® i601 (Roche Diagnostics, Mannheim, Germany) was evaluated in this study. The Ionify Immunosuppressants (iISD) reagent kit was used for the measurement of four immunosuppressants: tacrolimus, everolimus, cyclosporine, and sirolimus. This kit includes two independent components: 1) an internal standards (ISTD) flask containing ascomycin (45 ng/mL) and stable-isotope-labeled everolimus, cyclosporine, and sirolimus (990 ng/mL) in acetonitrile and 2) paramagnetic particles in suspension (50 mg/dL), which are used during the automatic sample preparation process within the analyzer. The analyzer was calibrated and controlled with calibrators and controls supplied by Roche (Ionify CalSet ISD and Ionify ControlSet ISD). Calibration was based on a predefined master calibration curve established by the manufacturer using an extended multipoint calibration. This master curve was adjusted using a two-point calibrator set, with analyte-specific concentrations provided by the manufacturer (level 1: 85.0 ng/mL, 1.50 ng/mL, 2.00 ng/mL, and 2.00 ng/mL; level 2: 1300 ng/mL, 17.0 ng/mL, 26.0 ng/mL, and 26.0 ng/mL of cyclosporine, everolimus, sirolimus, and tacrolimus, respectively). For the pretreatment of whole blood controls, calibrators, and samples, the Ionify Pretreatment Whole Blood (IPWB) was used, which lyses blood cells, releases the analytes, and precipitates the majority of blood proteins prior to centrifugation. The Cobas® i601 employs manufacturer-provided high-performance liquid chromatography (HPLC) cartridges based on reversed-phase liquid chromatography; as many as three parallel liquid chromatography streams can be configured, enabling a result output approximately every 2 min per stream, with a total assay run time of 34 min per analyte from sample loading to result generation. The analytical measurement ranges (AMRs) are tacrolimus 0.5–30 ng/mL, everolimus 0.5–20 ng/mL, sirolimus 0.5–30 ng/mL, and cyclosporine 10–1500 ng/mL.

Tacrolimus, everolimus, and cyclosporine results were compared with the immunoassay from Dimension® EXL™ 200 (Siemens Healthineers, Erlangen, Germany) from our routine laboratory (based on Affinity Chrome-Mediated Immunoassay [ACMIA] technology), and sirolimus, which is analyzed in an external laboratory, was compared to a manual LC–MS/MS system. The latter was performed using the commercial RECIPE ClinMass® TDM Kit System for Immunosuppressants (RECIPE Chemicals + Instruments, Munich, Germany), which complies with the European In Vitro Diagnostic Regulation (IVDR, EU 2017/746). The kit includes isotopically labeled internal standards for each analyte: d_12_-cyclosporine A, ^13^Cd_2_-tacrolimus, ^13^C_2_d_4_-everolimus, and ^13^Cd_3_-sirolimus. The assay was performed according to the manufacturer’s Instructions for Use (IFU). Sirolimus measurements were carried out on an Acquity UPLC I-Class system coupled to a Xevo TQ-S Micro tandem mass spectrometer (Waters, Milford, MA, USA), operating in positive electrospray ionization mode. Reverse-phase liquid chromatography was used. General source conditions included a capillary voltage of 3 kV, source temperature of 150 °C, desolvation temperature of 400 °C, desolvation gas flow of 1000 L/h, and cone gas flow of 100 L/h. The AMRs of the comparative methods were as follows: for the immunoassays performed on the Dimension® EXL™ 200 analyzer, cyclosporine 25–500 ng/mL, tacrolimus 1.0–30.0 ng/mL, and everolimus 1.0–25.0 ng/mL; and for sirolimus 0.30–93.1 ng/mL in the manual LC–MS/MS method.

### 2.2. Specimens

For the EP10-A3 evaluation [10], quality-control material Multichem MS ISDs (lot 90015900_90016000) from Technopath Clinical Diagnostics (Ballina, Ireland) was used. Each analyte was tested at three different levels: low, intermediate (a 1:1 mixture of low and high levels), and high. For the method comparison, aliquots from K_3_EDTA whole blood routine samples for immunosuppressants were collected. Samples were selected to cover a wide range of values across the Cobas i601 AMR. Pretreatment of calibrators, controls, and samples was performed according to manufacturer IFUs: 450 µL of calibrator, control, or whole blood sample was mixed with 50 µL of ISTD and 400 µL of IPWB, followed by vigorous mixing with a vortex for at least 10 s. After centrifugation of the samples (4 min, ≥10 000 *g*), the supernatant was introduced to the Cobas i601 analyzer. The sample preparation for sirolimus measurement at the external laboratory followed the manufacturer’s protocol. Briefly, 100 µL of whole blood was mixed with 20 µL of internal standard and 200 µL of precipitation reagent. The mixture was vortexed for 30 s, incubated at room temperature for 5 min, vortexed again for 10 s, and centrifuged at 10 000 *g* for 5 min. The resulting supernatant was transferred into an autosampler vial for LC–MS/MS injection.

Because we used only fully anonymized patient samples not obtained specifically for use in this study through an interaction or intervention with living individuals, neither informed consent nor IRB review was required.

### 2.3. Evaluation of Imprecision, Bias, and Linearity

A preliminary verification of imprecision, bias, and linearity was performed according to the CLSI EP10-A3 protocol [10]. Each quality-control level was analyzed in a specific sequence on five consecutive working days. Imprecision (coefficient of variation, CV; obtained with the mean and the standard deviation, SD), bias, and linearity were calculated using Analyse-it for Microsoft Excel, version 6.15.4 (Analyse-it Software, Leeds, UK). Linearity was assessed using simple linear regression between expected and measured concentrations. The obtained performance data were compared to our analytical performance specifications (APS), with a total analytical error (TE_A_) goal of ≤15% set on the basis of the recommendations of the International Association for Therapeutic Drug Monitoring and Clinical Toxicology (IATDMCT) [11]. The allowable CV and bias for each technique were set according to the equation TE_A_ = 1.65 × CV_A_ + B_A_, where CV_A_ is the analytical coefficient of variation and B_A_ is the analytical bias [12]. Results were also compared with the manufacturer’s claims in the IFU, where the precision results from the EP05‑A3 protocol are listed, performed with two levels of control (Multichem MS ISD). In addition, long-term cumulative imprecision data for the Dimension® EXL™ 200 immunoassays were obtained from our internal quality-control program using the Unity Real Time™ software (Bio-Rad Laboratories).

### 2.4. Method Comparison and Bias Estimation

To assess the interchangeability and bias between techniques, each test with the new Cobas i601 was compared to the test performed in our routine laboratory Dimension EXL 200 according to the CLSI EP09-A3 protocol [13]. Sirolimus results were compared to those obtained by an external laboratory performing LC–MS/MS. Patient samples were pretreated, and the supernatant was analyzed using the Cobas i601. Single measurements were carried out with the Dimension EXL 200 and with LC–MS/MS in an external laboratory, along with duplicate measurements with the Cobas i601, using 40 samples for each analyte. Samples from the Dimension EXL 200 were analyzed on the same day, whereas sirolimus samples were refrigerated (at 4 °C) and analyzed on the day of receipt or the following day at the external laboratory. Regression studies were performed using Analyse-it for Microsoft Excel (v6.15.4) following CLSI EP09-A3 recommendations. Simple Deming regression was applied when the differences between methods were constant in absolute terms (constant SD). Weighted Deming regression or Passing–Bablok regression was used when the differences were proportional to concentration (constant CV); weighted Deming was applied if parametric assumptions were met, whereas Passing–Bablok was used for non-normally distributed differences or in the presence of outliers. Predicted bias values relative to the Cobas i601 were determined from the regression equations at different concentrations considered as therapeutic-range concentrations (TRCs) and were compared to the allowable bias. According to CLSI recommendations, the confidence interval (CI) for the predicted bias of the Cobas i601 including or overlapping the allowable bias indicates that the method´s bias is not different from the allowable limit. Consequently, both methods could be considered interchangeable. We also considered the recommendations from the IATDMCT for acceptability of the comparison: a linear regression slope within ±10% of the theoretical value of 1.0, and a linear regression intercept not significantly different from zero, assessed in this study by examining the 95% confidence intervals obtained by the regression analysis [11]. Difference plots were generated as Bland–Altman plots representing the relative differences (%) between methods versus the mean of the two methods. The mean relative bias and the 95% limits of agreement (LoA) were calculated as the mean bias ± 1.96 standard deviations of the differences.

## 3. Results

### 3.1. Precision, Bias, and Linearity

The EP10-A3 evaluation results are presented in Table 1, and difference plots are shown in Figure 1. All four assays demonstrated good precision, in agreement with the EP05-A3 performance characteristics reported in Roche’s IFU. For every analyte, both the imprecision estimates and the calculated bias remained within the allowable APS defined in our laboratory. The non-linearity component was also consistently below the predefined APS limits. No analyte showed statistically significant deviation from linearity (*p* > 0.05), supporting the finding that the assays behaved linearly throughout the concentration ranges evaluated. Long-term cumulative imprecision for the Dimension EXL 200 immunoassays, calculated from internal quality-control data, was 9% for tacrolimus, 8.5% for everolimus, and 7% for cyclosporine, all of which are higher than the corresponding values obtained for the Cobas i601.

**Figure 1.**
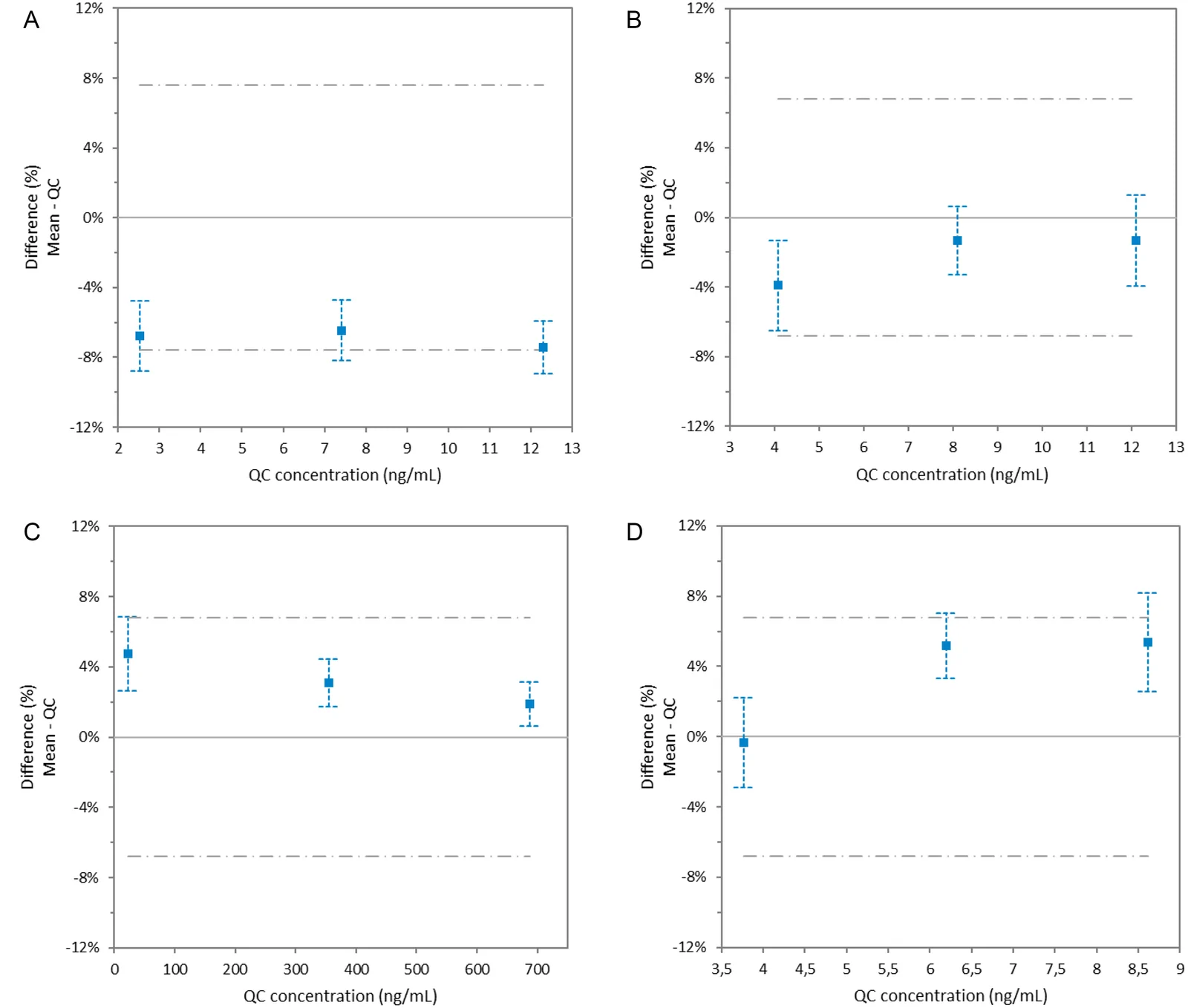
EP10-A3 difference plots showing relative bias (with 95% CI) for quality-control levels of A) tacrolimus, B) everolimus, C) cyclosporine, and D) sirolimus. Dashed lines represent the allowable difference limits.

**Table 1.** Precision, bias, and linearity verification results for immunosuppressants quantification on the Cobas i601 according to the CLSI EP10-A3 protocol, compared with analytical performance specifications and IFU results for EP05-A3.

Analyte, units	QC level		EP10-A3 results				APS goals^b^		IFU (EP05-A3)	
			Mean	CV (%)	Bias (%)	Non-linearity (%)^a^	CV (%)	Bias (%)	Mean	CV (%)
Tacrolimus, ng/mL	Low	2.52	2.35	3.9	-6.8	-0.9	4.5	7.6	2.43	5.4
	Medium	7.41	6.93	4.0	-6.5	0.6			—	—
	High	12.3	11.4	3.0	-7.4	-0.2			12.3	4.8
Everolimus, ng/mL	Low	4.09	3.93	4.7	-3.9	-0.4	5.0	6.8	3.76	5.4
	Medium	8.10	7.99	4.2	-1.3	0.4			—	—
	High	12.1	11.9	4.7	-1.3	-0.1			11.4	6.5
Cyclosporine, ng/mL	Low	23	24.1	3.6	4.7	-5.2	5.0	6.8	24.4	5.3
	Medium	355	366	2.8	3.1	0.7			—	—
	High	687	700	2.2	1.9	-0.2			665	6.6
Sirolimus, ng/mL	Low	3.77	3.76	4.4	-0.3	-0.8	5.0	6.8	3.89	5.6
	Medium	6.20	6.52	3.8	5.2	1.0			—	—
	High	8.62	9.08	4.8	5.4	-0.3			13.1	7.5

Abbreviations: QC, quality control; CV, coefficient of variation; APS, analytical performance specifications; IFU, instructions for use

a All tests showed a non-significant deviation from linearity (*p* > 0.05)

b Allowable CV (%) and allowable bias (%) calculated from a TE_A_ = 15% (TE_A_ = 1.65 × CV_A_ + B_A_) based on the recommendations of the International Association for Therapeutic Drug Monitoring and Clinical Toxicology (IATDMCT).

### 3.2. Method Comparison and Bias Estimation

The EP09-A3 comparison evaluation is summarized in Table 2, which includes Passing–Bablok regression equations and the TRC selected to evaluate the differences between methods. Figure 2 illustrates the regression analyses obtained for the comparison between the Dimension EXL 200 and the Cobas i601 for tacrolimus, everolimus, and cyclosporine, as well as those obtained for the comparison between the Cobas i601 and a manual LC–MS/MS method for sirolimus. Bland–Altman plots are depicted in Figure 3. Representative extracted ion chromatograms displayed by the Cobas i601 system for the evaluated immunosuppressants are shown in Supplementary Figure 1.

**Figure 2.**
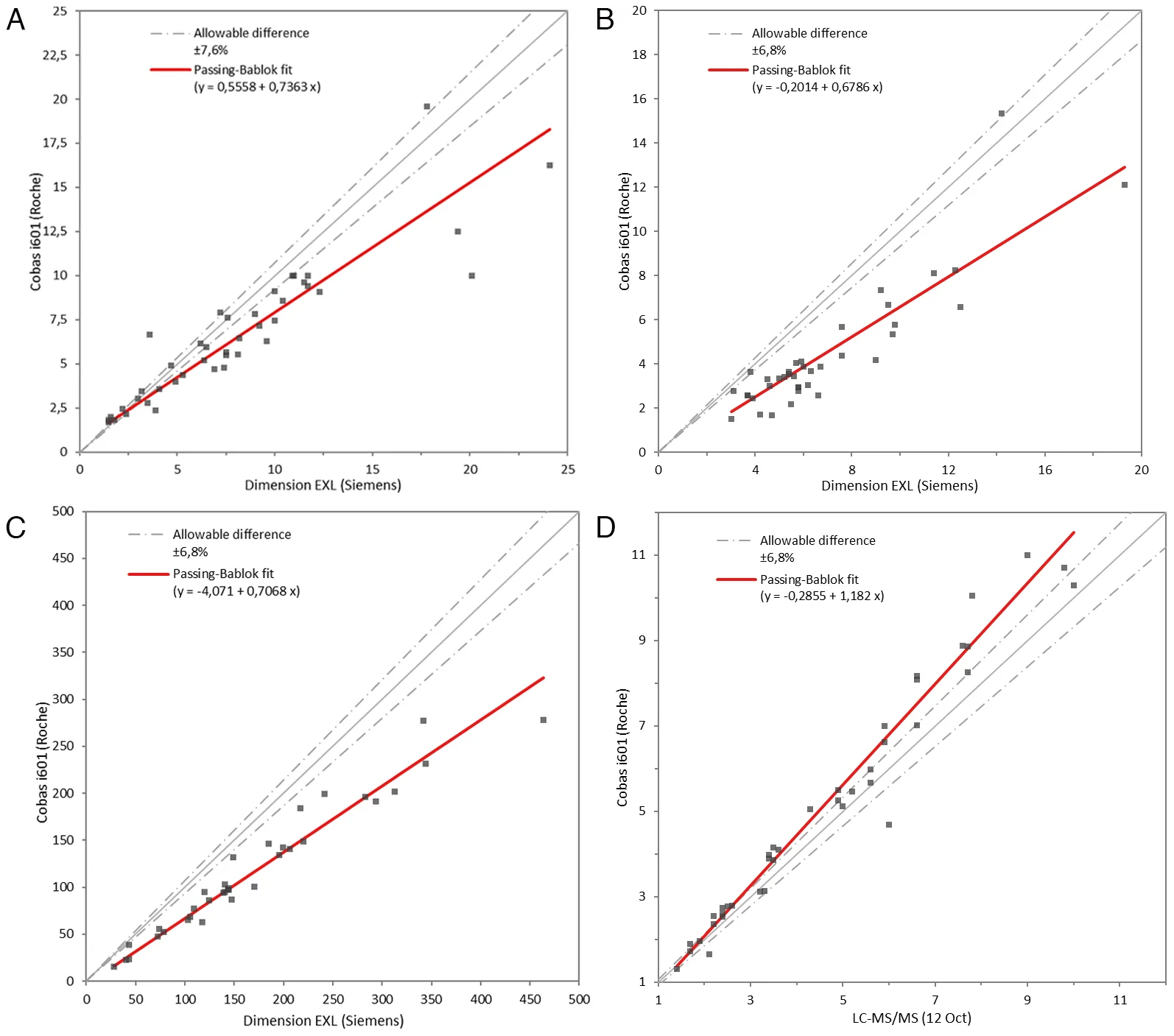
Passing–Bablok regression results from the method comparison according to the EP09-A3 protocol: A) tacrolimus (ng/mL), B) everolimus (ng/mL), C) cyclosporine (ng/mL), and D) sirolimus (ng/mL). Dashed lines represent the allowable difference limits.

**Figure 3.**
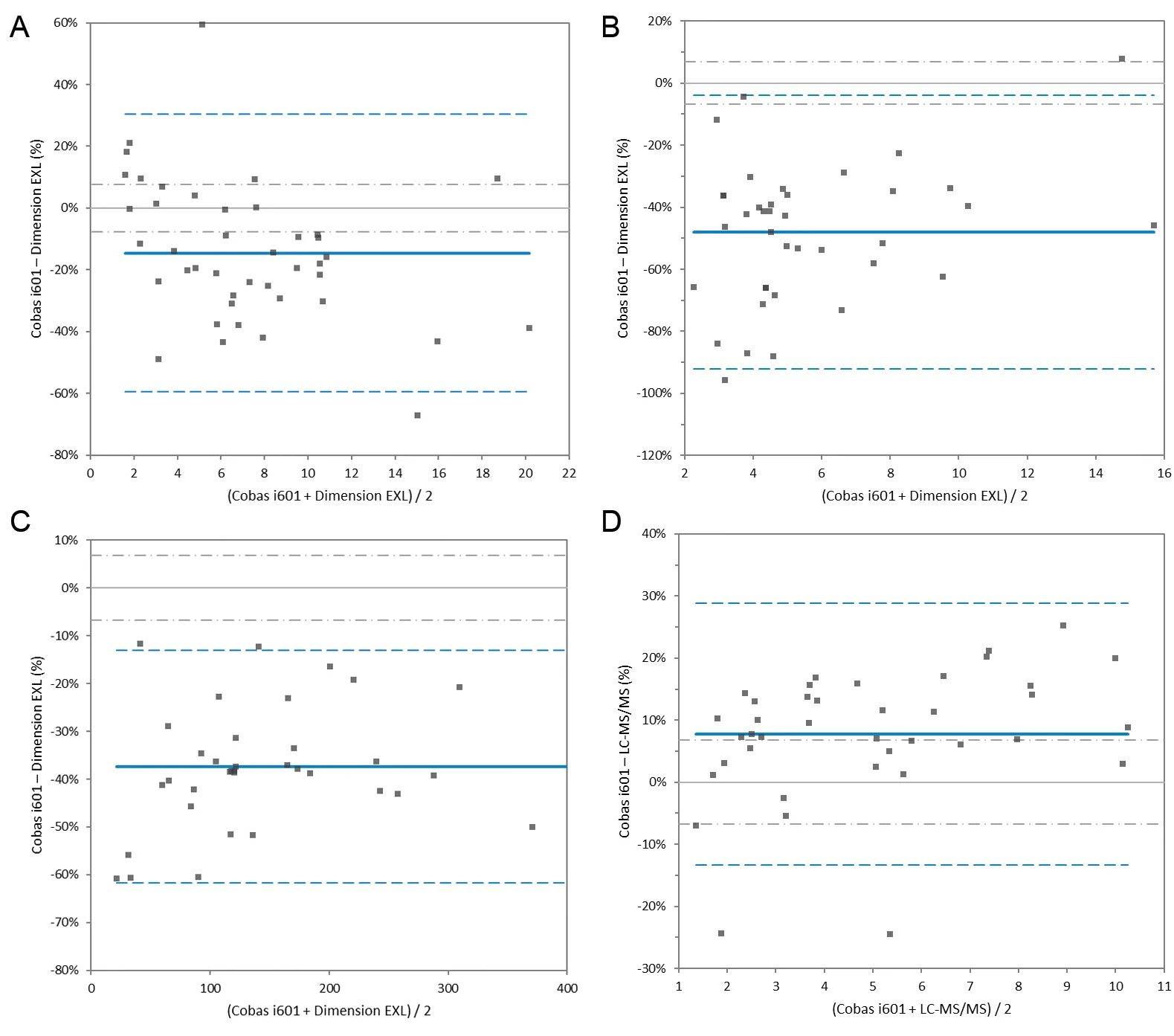
Bland-Altman difference plots from method comparison according to EP09-A3 protocol: A) tacrolimus (ng/mL), B) everolimus (ng/mL), C) cyclosporine (ng/mL), and D) sirolimus (ng/mL). Grey dashed lines represent the allowable difference limits based on analytical performance specifications. The solid blue line represents the mean relative bias, and the dashed blue lines represent the upper and lower 95% limits of agreement (LoA).

**Table 2.** Method comparison of four immunosuppressants on the Cobas i601 (LC-MS/MS) (Y) compared to Dimension EXL 200 (tacrolimus, everolimus, and cyclosporine) and a manual LC-MS/MS method for sirolimus (X) according to CLSI EP09-A3 protocol, and therapeutic range concentrations with mean difference associated.

Analyte, units	Slope^a^ (95% CI)	Intercept^a^ (95% CI)	Correlation (*r*)	Range tested in Cobas i601	TRC	Mean difference(%)	95% CI (%)	Allowable difference (APS)^b^ (%)	EP09-A3 interpretation^c^
Tacrolimus, ng/mL	**0.736** (0.651 to 0.854)	**0.556** (0.017 to 0.097)	0.910	1.62-20.3	4	-12.5	(-17.5 to -4.6)	7.6	-
					8	-19.4	(-24.0 to -12.2)		
					15	-22.7	(-28.9 to -13.8)		
Everolimus, ng/mL	**0.679** (0.540 to 0.865)	-0.201 (-1.498 to 0.560)	0.895	1.49-15.7	3	-38.90	(-66.7 to -27.3)	6.8	-
					8	-34.70	(-42.2 to -29.9)		
Cyclosporine^d^, ng/mL	**0.707** (0.644 to 0.789)	-4.071 (-10.97 to 5.240)	0.973	14.8-278	50	-37.5	(-45.4 to -23.9)	6.8	-
					100	-33.4	(-35.3 to -29.5)		
					400	-30.3	(-34.4 to -24.0)		
Sirolimus, ng/mL	**1.182** (1.098 to 1.267)	**-0.286** (-0.513 to -0.034)	0.981	1.3-11.3	5	12.5	(7.4 to 17.5)	6.8	-
					15	16.3	(9.3 to 23.6)		
					20	16.8	(9.5 to 24.4)		

Abbreviations: TRC, therapeutic range concentrations; CI, confidence interval; APS, analytical performance specifications

a Bold numbers indicate slopes with a CI that does not include 1 and intercepts with an interval coefficient that does not include 0.

b Allowable difference calculated based on recommendations of the International Association for Therapeutic Drug Monitoring and Clinical Toxicology (IATDMCT).

c Following the CLSI recommendations from EP09-A3 protocol: (+) methods interchangeable at the CDL (allowable bias includes or overlaps the CI for the predicted bias); (-) methods not interchangeable (allowable bias is less than the absolute value of the lower limit of the CI of the predicted bias).

d For cyclosporine, three samples exceeding the analytical measurement range of the Dimension EXL 200 immunoassay (>500 ng/mL) were excluded from the regression analysis to comply with CLSI EP09-A3 recommendations.

For tacrolimus, everolimus, and cyclosporine, concentrations measured with the Cobas i601 were lower than those measured with the Dimension EXL 200 in most samples. The correlation coefficient (*r*) was higher for cyclosporine and lower for tacrolimus and everolimus. For all three analytes, regression slopes were below 1 and lower than 0.9 (10% of the 1.0 acceptance criteria from IATDMCT). The 95% CI for the slopes did not include 1, and the intercept CI included 0 except in the case of tacrolimus, indicating the presence of proportional and systematic differences between the methods (Table 2). On the basis of the predefined TRC, the mean percentage differences between both methods were larger for everolimus and cyclosporine (approximately –35%) and smaller for tacrolimus (–12.5% at 4 ng/mL and –22.7% at 15 ng/mL). These differences exceeded our predefined allowable bias specifications for all analytes except at the lowest tacrolimus concentration evaluated (4 ng/mL), where the 95% CI (–17.5% to –4.6%) overlapped the allowable bias limit of –7.6%. For cyclosporine, three samples exceeding the AMR of the Dimension EXL 200 immunoassay (>500 ng/mL) were excluded from the regression analysis, in accordance with CLSI EP09-A3 recommendations.

By contrast, the sirolimus concentrations determined using the Cobas i601 were higher than those determined by the LC–MS/MS method. The regression slope was greater than 1.1, exceeding the 10% deviation from unity, according to the IATDMCT criteria. Theslope CI did not include 1, and the intercept CI did not include 0 (Table 2), indicating systematic differences. Although the calculated bias using TRC exceeded the predefined allowable limit, sirolimus showed the smallest overall mean bias among the investigated immunosuppressants.

Across all four immunosuppressants, the magnitude of the differences increased with increasing concentration, indicating a proportional bias rather than a constant systematic offset. We therefore constructed Bland–Altman plots (Figure 3) using relative (%) differences to provide a more appropriate visualization of agreement across the analytical range. The mean relative bias was –14.6% for tacrolimus (95% LoA: –58.8% to 29.6%), –48.0% for everolimus (LoA: –91.4% to –4.6%), and –37.6% for cyclosporine (LoA: –62.4% to –12.8%). For sirolimus, the mean relative bias was +7.8% (LoA: –12.3% to 27.9%). Everolimus and cyclosporine exhibited a marked negative proportional bias across the measurement range, consistent with the regression findings and the concentration-dependent differences observed at TRCs. Tacrolimus showed a moderate negative bias with wide limits of agreement, indicating substantial dispersion of individual differences. Compared with the other immunosuppressants, sirolimus showed the smallest overall mean positive bias and narrower limits of agreement.

## 4. Discussion

In this study, we evaluated the analytical performance of the Cobas i601, an automated LC–MS/MS platform from Roche Diagnostics, for the quantification of the immunosuppressive drugs tacrolimus, cyclosporine, everolimus, and sirolimus in whole blood. In addition to assessing precision, bias, and linearity, we compared the results obtained with the Cobas i601 to those generated by the immunoassays currently used in our clinical laboratory, with the aim of understanding the potential impact on TDM when transitioning between technologies.

The Cobas i601 exhibited good analytical precision, aligned with the manufacturer’s specifications and within the predefined allowable limits. Bias and linearity assessments also yielded satisfactory outcomes, confirming reliable analytical behavior across the measurement range. When compared with the Dimension EXL 200, the Cobas i601 demonstrated superior overall performance, reflected by the higher long-term cumulative imprecision obtained with an internal quality-control material (tacrolimus 9%, everolimus 8.5%, cyclosporine 7%). These findings underscore the analytical robustness of the system and support its suitability for routine TDM of immunosuppressive drugs.

For tacrolimus, the Cobas i601 produced concentrations generally lower than those obtained with the Dimension EXL 200. At low concentrations (e.g., 4 ng/mL), the bias (–12.5%; 95% IC –17.5% to –4.6%) included the allowable bias (±7.6%). However, at higher concentrations, the bias obtained increased (i.e., –19.4% at 8 ng/mL and –22.7% at 15 ng/mL) and the CIs no longer overlapped the acceptable bias. This result is further supported by the wide LoA observed in the Bland–Altman analysis, indicating substantial variability at the individual sample level. Therefore, the Cobas i601 and Dimension EXL 200 methods are not interchangeable for tacrolimus measurement. Previous studies comparing tacrolimus immunoassays and LC–MS/MS have showed positive bias for immunoassay techniques [14,15]. According to the Dimension EXL 200 IFU, metabolites such as 12-*O*-hydroxyl-tacrolimus (M-IV) and 13,15-*O*-didesmethyl-tacrolimus (M-VII) exhibit strong cross-reactivity (99% and 43% at 40 ng/mL, respectively), and additional studies have reported relevant cross-reactivity for other metabolites (e.g., 31-*O*-desmethyl-tacrolimus (M-II) and 15-*O*-desmethyl-tacrolimus (M-III)) higher than the IFU specified [16]. These interferences remain clinically important because tacrolimus dosing relies predominantly on trough concentrations with well-defined therapeutic windows (e.g., 4–12 ng/mL in kidney transplant recipients, covering both the early post-transplant and maintenance phases, as well as in highly sensitized patients) [17,18]. Even moderate negative bias may lead to misinterpretation of results if not properly communicated, reinforcing the need to reassess target ranges when adopting the new platform.

For everolimus, the Cobas i601 showed an even larger negative bias compared with the immunoassay, reaching –38.9% at 3 ng/mL and –34.7% at 8 ng/mL. The correlation coefficient was the lowest among the immunosuppressants tested, with a greater scatter of data points (Figure 2). The Bland–Altman analysis confirmed not only a marked negative mean bias but also very wide LoA, reinforcing the lack of interchangeability across the analytical range. This poorer agreement may reflect the known limitations of immunoassays for everolimus, which are prone to positive interference from drug metabolites and other matrix effects, leading to variable overestimation depending on patient metabolism and assay design. According to the Dimension EXL 200 IFU, cross-reactivity with sirolimus (particularly during treatment transition periods) and several everolimus metabolites (e.g., 25-hydroxy-everolimus, 12-hydroxy-everolimus) can occur. Such interferences and positive bias with immunoassays have been described previously in several studies comparing LC–MS/MS and immunoassay methods for everolimus quantification [19,20], reinforcing the need to interpret results carefully when transitioning between platforms. More specifically, a comparison between the ACMIA immunoassay from Siemens Healthineers and the Chromsystems MassTox LC–MS/MS method revealed a substantial positive bias (slope 1.511; intercept 0.244) [21], consistent with our findings. Given the narrow therapeutic window of everolimus and its use in transplantation and oncology, these systematic differences are clinically relevant and must be addressed through appropriate interpretative guidance when switching technologies.

In the case of cyclosporine, the bias observed compared with the Dimension EXL 200 results was similar to that in the case of everolimus (–37.5% at 50 ng/mL, –33.4% at 100 ng/mL, and –30.3% at 400 ng/mL), indicating that the methods are not interchangeable. However, cyclosporine showed the strongest correlation coefficient among the four immunosuppressors (*r* = 0.973), despite the substantial negative bias. This pattern suggests a systematic difference between methods rather than random variation, likely reflecting consistent overestimation by the immunoassay, combined with relatively low matrix interferences and a proportional response across the measurement range. Consistently, Bland–Altman analysis demonstrated a large negative mean bias with a relatively wide LoA, supporting the presence of clinically relevant proportional differences. Previous studies have also reported positive bias for immunoassays versus LC–MS/MS, and some have described organ-specific variation in the bias magnitude (e.g., 20.2% in liver transplant recipients vs. 9.1% in heart transplant recipients) [22]. Clinicians should be advised to treat a method change as a potential source of discontinuity in therapeutic monitoring, and guidance from transplantation protocols (which increasingly reference MS-based assays) should be consulted to adjust decision limits accordingly. An additional consideration in the cyclosporine comparison is the different AMR of the two methods (25–500 ng/mL for the immunoassay vs. 10–1500 ng/mL for the LC–MS/MS method). Consequently, the comparison was restricted to concentrations within the AMR of the immunoassay.

Interestingly, in the case of sirolimus, the Cobas i601 yielded higher concentrations than the manual LC–MS/MS method. Although the mean bias exceeded our allowable specification across all TRCs (±6.8%), we note that this threshold is particularly stringent and challenging (based on a TE_A_ goal of ≤15%), as highlighted in the IATDMCT recommendations [11]. The LoA were narrower than those observed for the other immunosuppressants; however, the presence of systematic differences indicates that direct interchangeability cannot be assumed without further evaluation. Moreover, sirolimus samples were not analyzed within the 2-h timeframe recommended by the CLSI EP09-A3 protocol; because of external processing, samples were measured several hours after collection or the following day. This deviation from contemporaneous analysis could theoretically introduce additional variability. However, according to the manufacturer’s IFU, whole blood sirolimus samples are stable for as long as 7 days at 2–8 °C. Because samples were transported and stored under refrigerated conditions within this validated stability window, it is unlikely that the time delay substantially affected the measured concentrations. The external laboratory reported a CV of 6% and negative bias of –4% (meeting the ≤15% TE_A_ goal), whereas the bias obtained with the EP10-A3 protocol for sirolimus was positive: 5.2% (at 6.2 ng/mL). We also note that the two methods differ in their metrological traceability: the Cobas i601 sirolimus assay is traceable to the Joint Committee for Traceability in Laboratory Medicine (JCTLM) reference measurement procedure C16RMP9R [23], which shows excellent agreement in method comparison (Deming regression: *y* = 0.997*x* + 0.0496; *r* = 0.994), whereas the external laboratory uses reference material from RECIPE. For these reasons, participation in external quality assessment (EQA) programs and comparison with a broader set of LC–MS/MS laboratories will help further consolidate the assessment of the sirolimus performance of the Cobas i601.

Transitioning from immunoassay to an automated LC–MS/MS platform such as the Cobas i601 provides substantial clinical and operational advantages. Beyond the expected improvements in analytical specificity and elimination of metabolite cross-reactivity, LC–MS/MS offers better alignment with current TDM recommendations, which increasingly emphasize mass-spectrometry-based quantification. These advantages are particularly relevant in clinical scenarios where precise measurement is critical, such as the early post-transplant period, in patients with high metabolite burden (e.g., fast metabolizers, pediatric recipients), or in the presence of strong CYP3A inhibitors or inducers [24,25]. In these settings, immunoassays tend to overestimate concentrations, whereas LC–MS/MS more accurately reflects pharmacokinetic and pathophysiological variability. From a biological standpoint, metabolite cross-reactivity is clinically meaningful because tacrolimus and everolimus metabolites can be substantially increased in CYP3A5 expressers, hepatic dysfunction, or drug–drug interactions (further supporting the rationale for adopting MS-based TDM). From an operational point of view, a fully automated LC–MS/MS platform offers important workflow benefits, including reduced hands-on time, simplified reagent handling, and minimal instrument downtime. Its seamless integration into routine laboratory systems increases throughput and shortens turnaround times, whereas consolidation of multiple assays on a single platform enhances overall productivity and robustness.

Despite these benefits, transitioning between analytical technologies inevitably introduces discontinuities in longitudinal patient data. Effective clinician communication, careful interpretation during the transition phase, and, when appropriate, revision of therapeutic ranges or inclusion of method-change alerts in laboratory reports are essential to avoid misclassification of patients as subtherapeutic, therapeutic, or supratherapeutic. Operationally, automated LC–MS/MS reduces manual workload, minimizes human error, and increases throughput, facilitating broader access to high-specificity measurement in routine clinical practice.

Among the limitations of this study, the non-contemporaneous analysis of sirolimus samples may have introduced additional variability even though samples were transported and stored under refrigerated conditions within the manufacturer stability window. The study was unicentric, and only a single lot of reagent kits was used. In addition, sirolimus was not compared with the immunoassay, nor were tacrolimus, everolimus, or cyclosporine assessed against a manual LC–MS/MS method; comparisons were performed according to the analytical methods available in our routine workflow, and more extensive cross-platform evaluations were not feasible within the operational context of the study. Furthermore, trueness was not assessed using commutable reference materials or reference measurement procedures; therefore, the bias estimates obtained in this study reflect method-dependent differences rather than metrological accuracy. Notably, the observed differences cannot be directly extrapolated to other immunoassays because each assay has intrinsic characteristics related to its methodology. Although the sample size for method comparison met CLSI EP09-A3 requirements for most analytes, fewer samples were included in the cyclosporine comparison after exclusion of results exceeding the AMR of the immunoassay. A larger dataset and long-term follow-up including clinical outcomes (such as the impact on dose-adjustment decisions) would further strengthen the reliability of these findings; however, correlating analytical differences with clinical outcomes was beyond the scope of this study and represents a valuable direction for future research. By contrast, a key strength of the study is that the evaluation was performed under real-world routine laboratory workflow conditions and followed standardized CLSI protocols, supporting the applicability of the results to everyday clinical practice.

## 5. Conclusion

The Cobas i601 automated LC–MS/MS system demonstrated good analytical precision and linearity for immunosuppressant quantification under the conditions evaluated, meeting the analytical performance specifications. Substantial method-dependent biases were observed for several analytes when compared with the results of immunoassays used in our laboratory, particularly for everolimus and cyclosporine, emphasizing that direct interchangeability between methodologies cannot be assumed, with biases exceeding –30% for these drugs. Nevertheless, the system shows strong potential for routine LC–MS/MS implementation in TDM in clinical laboratories, and our evaluation supports the verified analytical performance of the platform. Method transition must be carefully managed, and clinician communication, re-evaluation of therapeutic ranges, and appropriate implementation strategies are essential to avoid misinterpretation of patient results.

## Supplementary Material

Supplementary Material

## Data Availability

The data that support the findings of this study are available from the corresponding author upon reasonable request.

## Abbreviations

ACMIA: Affinity Chrome-Mediated Immunoassay
AMR: Analytical Measurement Range
APS: Analytical Performance Specifications
B_A_: Analytical Bias
CI: Confidence Interval
CLSI: Clinical Laboratory Standards Institute
CV: Coefficient of Variation
IATDMCT: International Association for Therapeutic Drug Monitoring and Clinical Toxicology
IFU: Instructions for Use
IPWB: Ionify Pretreatment Whole Blood
ISTD: Internal Standard
IVDR: In Vitro Diagnostic Regulation
LC–MS/MS: Liquid Chromatography–Tandem Mass Spectrometry
LoA: Limits of Agreement
SD: Standard Deviation
TDM: Therapeutic Drug Monitoring
TE_A_: Total Analytical Error
TRC: Therapeutic Range Concentration
