# Supplementary material for: Preliminary Analytical Evaluation of the Automated Cobas i601 LC-MS/MS System for Immunosuppressant Quantification in a Clinical Laboratory
Source: J Mass Spectrom Adv Clin Lab. 2026 May 19;39(1):rtbk5y18. doi: 10.21627/rtbk5y18 (PMC13750201; doi:10.21627/rtbk5y18)
Supplement: Supplementary file 1 [file jmsacl-39-1-rtbk5y18-s001.pdf]

SUPPLEMENTARY MATERIAL

Preliminary Analytical Evaluation of the Automated Cobas i601 LC-MS/MS System for Immunosuppressants Quantification in a Clinical Laboratory

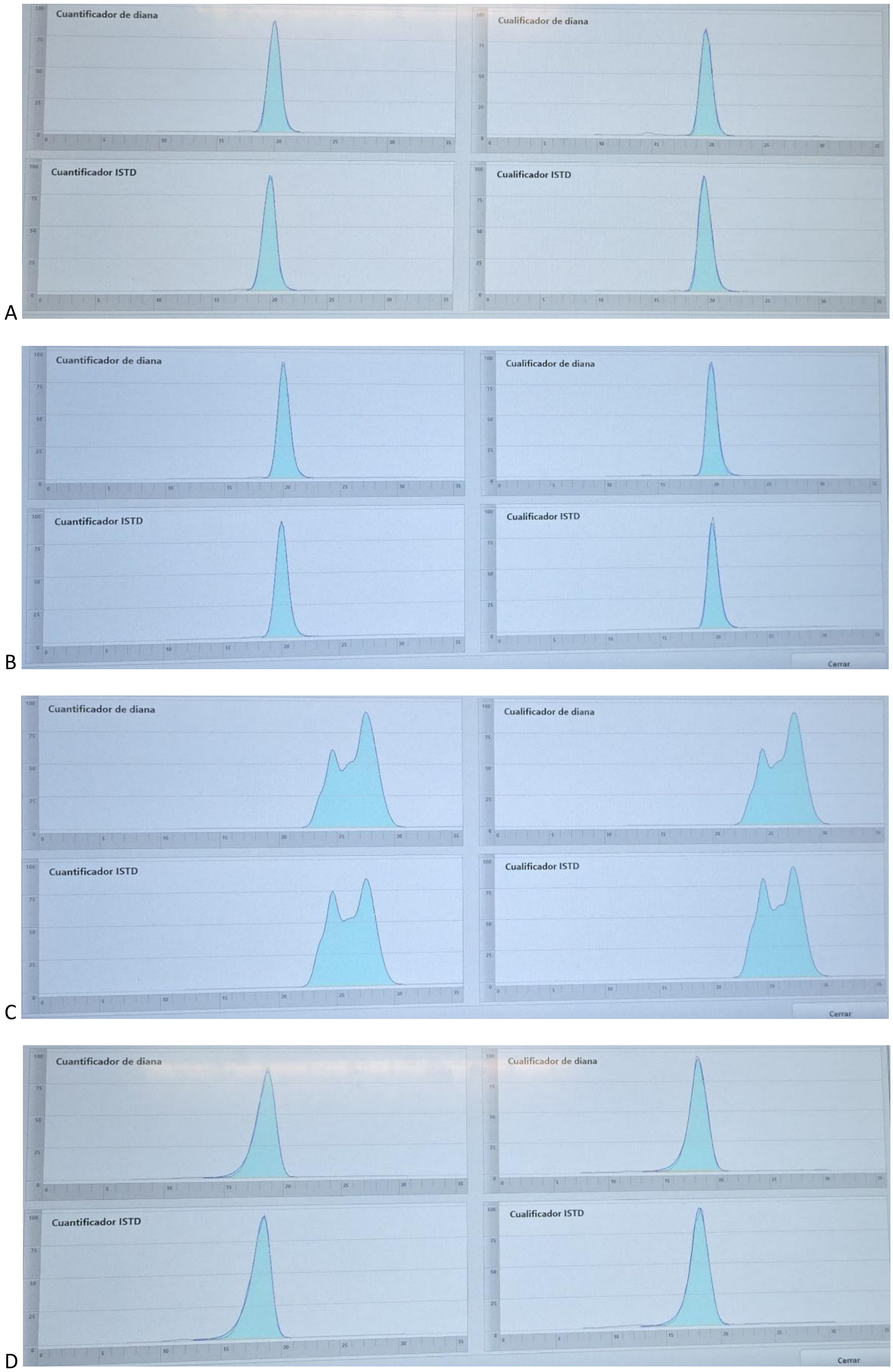

Supplementary Figure 1. Representative extracted ion chromatograms as displayed by the Cobas i601 system for A) sirolimus, B) everolimus, C) cyclosporine, and D) tacrolimus.
